# Draft genome sequence of marine-derived *Streptomyces* sp. TP-A0598, a producer of anti-MRSA antibiotic lydicamycins

**DOI:** 10.1186/s40793-015-0046-5

**Published:** 2015-08-26

**Authors:** Hisayuki Komaki, Natsuko Ichikawa, Akira Hosoyama, Nobuyuki Fujita, Yasuhiro Igarashi

**Affiliations:** Biological Resource Center, National Institute of Technology and Evaluation (NBRC), Chiba, Japan; NBRC, Tokyo, Japan; Biotechnology Research Center and Department of Biotechnology, Toyama Prefectural University, Toyama, Japan

**Keywords:** Lydicamycin, TPU-0037, Biosynthetic gene, Polyketide synthase, *Streptomyces*

## Abstract

*Streptomyces* sp. TP-A0598, isolated from seawater, produces lydicamycin, structurally unique type I polyketide bearing two nitrogen-containing five-membered rings, and four congeners TPU-0037-A, −B, −C, and –D. We herein report the 8 Mb draft genome sequence of this strain, together with classification and features of the organism and generation, annotation and analysis of the genome sequence. The genome encodes 7,240 putative ORFs, of which 4,450 ORFs were assigned with COG categories. Also, 66 tRNA genes and one rRNA operon were identified. The genome contains eight gene clusters involved in the production of polyketides and nonribosomal peptides. Among them, a PKS/NRPS gene cluster was assigned to be responsible for lydicamycin biosynthesis and a plausible biosynthetic pathway was proposed on the basis of gene function prediction. This genome sequence data will facilitate to probe the potential of secondary metabolism in marine-derived *Streptomyces*.

## Introduction

**Fig. 1:**
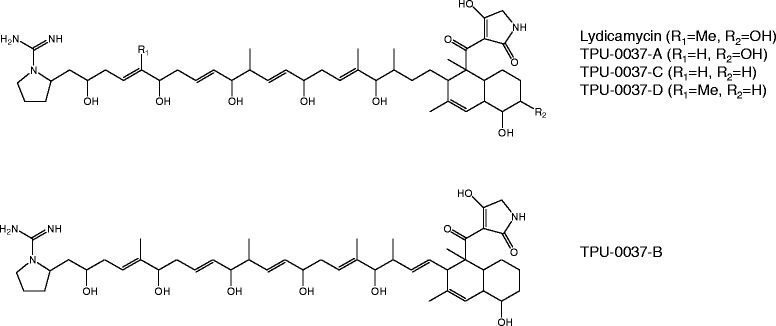
Chemical structures of lydicamycin and its congeners produced by *Streptomyces* sp. TP-A0598

Members of the genus *Streptomyces*, Gram-positive filamentous actinomycetes, are an attractive source for bioactive secondary metabolites. Terrestrial surface soil is the most common habitat for *Streptomyces* but a recent survey has disclosed its ubiquitous distribution in marine environments. Marine *Streptomyces* are currently attracting much attention as an untouched resource of novel bioactive compounds useful for drug development [1–3]. In our screening for new anti-MRSA antibiotics, *Streptomyces* sp. TP-A0598 collected from deep sea water was found to produce lydicamycin and its four new congeners of polyketide origin (Fig. 1) [4]. Lydicamyicn is characterized by the unprecedented pyrrolidine ring modified by an aminoiminomethyl group to which a polyketide-derived carbon chain with multiple hydroxyl and olefinic functionalities is linked and to the other end of the chain is linked an octalin modified by a tetramic acid. Despite this unique structural feature, biosynthetic genes of lydicamycin have not been reported to date. In this study, we conducted whole genome shotgun sequencing of the strain TP-A0598 to identify the PKS gene cluster for lydicamycin. We herein present the draft genome sequence of *Streptomyces* sp. TP-A0598, together with the description of genome properties and annotation for secondary metabolite genes. The putative lydicamycin biosynthetic gene cluster and a plausible biosynthetic pathway are also reported.

## Organism information

### Classification and features

**Fig. 2:**
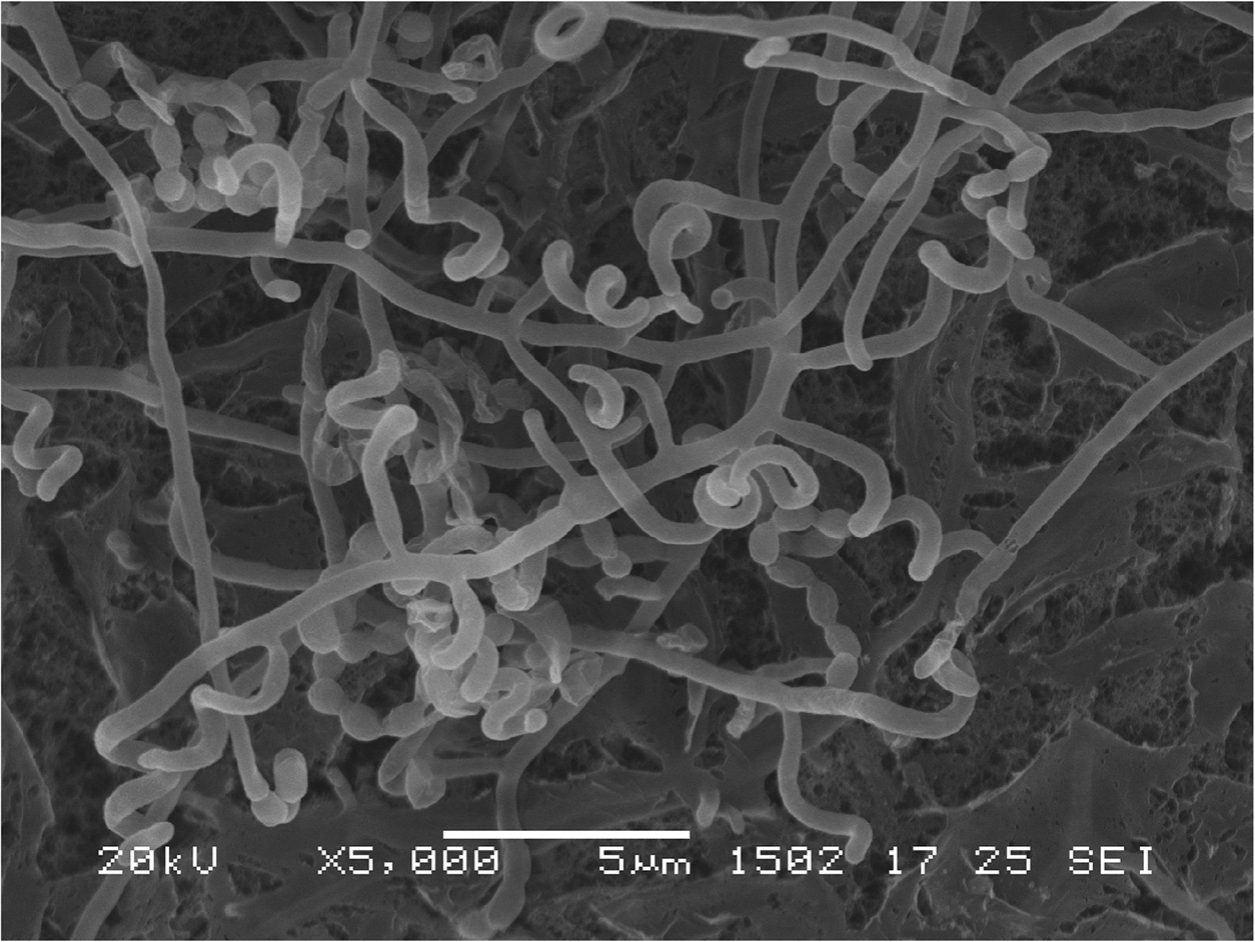
Scanning electron micrograph of *Streptomyces* sp. TP-A0598 grown on ten-fold diluted ISP 2 medium agar for 11 days at 28 °C. Bar, 5 μm

**Fig. 3:**
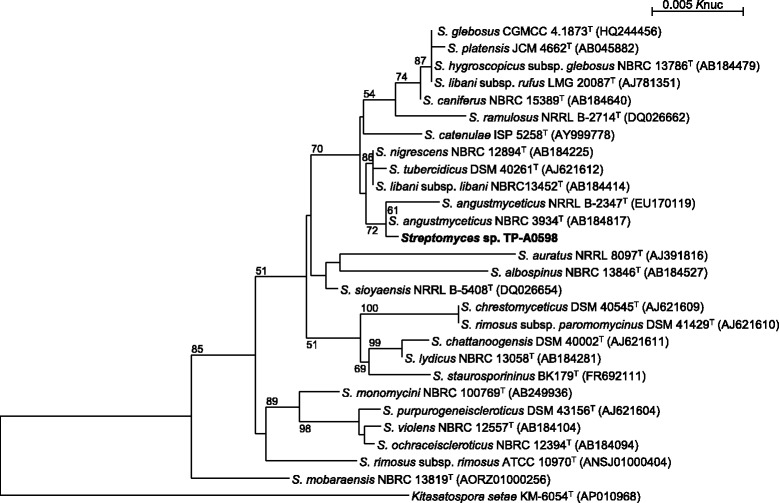
Phylogenetic tree highlighting the position of *Streptomyces* sp. TP-A0598 relative to phylogenetically close type strains within the genus *Streptomyces.* The strains and their corresponding GenBank accession numbers for 16S rRNA genes are shown in parentheses. The tree uses sequences aligned by ClustalX2 [8], and constructed by the neighbor-joining method [27]. All positions containing gaps were eliminated. The building of the tree also involves a bootstrapping process repeated 1000 times to generate a majority consensus tree [28], and only bootstrap values above 50 % are shown at branching points. *Kitasatospora setae* [29] was used as an outgroup

In the course of screening for new bioactive molecules produced by marine microorganisms, *Streptomyces* sp. TP-A0598 was isolated from a seawater sample collected in 2,600 meters off the shore and 321 meters in depth at Namerikawa, Toyama, Japan by a membrane filter method and found to produce lydicamycin and its novel congeners. This strain grew well on Bennett’s, ISP 3, ISP 4, ISP 5 and Yeast starch agars. On ISP 5, ISP 6 and ISP 7 agars, the growth was poor. The color of aerial mycelia was grayish olive and that of the reverse side was pale yellow on ISP 3 agar. Diffusible pigments were not formed on any agar media that we examined. Strain TP-A0598 formed spiral spore chains and the spores were cylindrical, 0.5 × 0.9 μm in size, having a warty surface [4]. A scanning electron micrograph of this strain is shown in Fig. 2. Growth occurred at 15–37 °C (optimum 30 °C) and pH 5–9 (optimum pH 7). Strain TP-A0598 exhibited growth with 0–7 % (w/v) NaCl (optimum 0 % NaCl). Strain TP-A0598 utilized D-glucose, sucrose, inositol, L-rhamnose, D-mannitol, D-raffinose, D-fructose, L-arabinose, and D-xylose for growth (Table 1) [4]. This strain was deposited in the NBRC culture collection with the registration number of NBRC 110027. The genes encoding 16S rRNA were amplified by PCR using two universal primers, 9 F and 1541R. After purification of the PCR product by AMPure (Beckman Coulter), the sequencing was carried out according to a established methods [5]. Homology search of the sequence by EzTaxon-e [6] indicated the highest similarity (99.93 %, 1465/1466) to *Streptomyces angustmyceticus*NBRC 3934^T^ (AB184817) [7] as the closest type strain. A phylogenetic tree was reconstructed on the basis of the 16S rRNA gene sequence together with phylogenetic neighbors that showed over 98.5 % similarity (Fig. 3) using ClustalX2 [8] and NJplot [9]. The phylogenetic analysis confirmed that the strain TP-A0598 belongs to the genus *Streptomyces*.

**Table 1:** Classification and general features of *Streptomyces* sp. TP-A0598

MIGS ID	Property	Term	Evidence code^a^
	Classification	Domain *Bacteria*	TAS [16]
		Phylum *Actinobacteria*	TAS [17]
		Class *Actinobacteria*	TAS [18]
		Order *Actinomycetales*	TAS [18–21]
		Suborder *Streptomycineae*	TAS [18, 19]
		Family *Streptomycetaceae*	TAS [18–20, 22, 23]
		Genus *Streptomyces*	TAS [20, 23–25]
		Species *Streptomyces* sp.	TAS [4]
		Strain TP-A0598	TAS [4]
	Gram stain	Not tested, likely positive	NAS
	Cell shape	Branched mycelia	TAS [4]
	Motility	Not reported	
	Sporulation	Sporulating	TAS [4]
	Temperature range	Grows from 15 °C to 37 °C	IDA
	Optimum temperature	30 °C	IDA
	pH range; Optimum	5-9; 7	IDA
	Carbon source	D-glucose, sucrose, inositol, L-rhamnose, D-mannitol, D-raffinose, D-fructose, L-arabinose, D-xylose	TAS [4]
MIGS-6	Habitat	Marine	TAS [4]
MIGS-6.3	Salinity	Grows from 0 % to 7 % NaCl	IDA
MIGS-22	Oxygen requirement	Aerobic	TAS [4]
MIGS-15	Biotic relationship	Free-living	TAS [4]
MIGS-14	Pathogenicity	Not reported	
MIGS-4	Geographic location	2,600 meters off the shore at Namerikawa, Toyama, Japan	TAS [4]
MIGS-5	Sample collection	Not reported	
MIGS-4.1	Latitude	Not reported	
MIGS-4.2	Longitude	Not reported	
MIGS-4.4	Attitude	−321 m	TAS [4]

^a^Evidence codes – IDA: Inferred from Direct Assay; TAS: Traceable Author Statement (i.e., a direct report exists in the literature); NAS: Non-traceable Author Statement (i.e., not directly observed for the living, isolated sample, but based on a generally accepted property for the species, or anecdotal evidence). These evidence codes are the Gene Ontology project [26]

### Chemotaxonomic data

The whole-cell hydrolysates of strain TP-A0598 contained L,L-diaminopimelic acid, glycine, ribose and madurose. The cellular fatty acids consisted of 21 % 14-methylpentadecanoic acid (*iso* C_16_), 9 % 13-methyltetradecanoic acid (*iso* C_15:0_), 8 % 12-methyltetradecanoic acid (*anteiso* C_15:0_) and other minor fatty acids [4].

## Genome sequencing information

### Genome project history

In collaboration between Toyama Prefectural University and NBRC, the organism was selected for genome sequencing to elucidate the lydicamycin biosynthetic gene cluster. We successfully accomplished the genome project of *Streptomyces* sp. TP-A0598 as reported in this paper. The draft genome sequence data have been deposited in the INSDC database under the accession number BBNO01000001-BBNO01000020. The project information and its association with MIGS version 2.0 compliance are summarized in Table 2 [10].

**Table 2:** Project information

MIGS ID	Property	Term
MIGS 31	Finishing quality	Improved-high-quality draft
MIGS-28	Libraries used	454 shotgun library, Illumina pair-end library
MIGS 29	Sequencing platforms	454 GS FLX+, Illumina HiSeq1000
MIGS 31.2	Fold coverage	8.4 ×, 93 ×, respectively
MIGS 30	Assemblers	Newbler v2.6
MIGS 32	Gene calling method	Progidal v2.6
	Locus Tag	TPA0598
	GenBank ID	BBNO00000000
	GenBank Date of Release	January 6, 2015
	GOLD ID	Not registered
	BIOPROJECT	PRJDB3150
MIGS 13	Source Material Identifier	NBRC 110027
	Project relevance	Industrial

### Growth conditions and genomic DNA preparation

*Streptomyces* sp. TP-A0598 monoisolate was grown on polycarbonate membrane filter (Advantec) on double diluted ISP 2 agar medium (0.2 % yeast extract, 0.5 % malt extract, 0.2 % glucose, 2 % agar, pH 7.3) at 28 °C. High quality genomic DNA for sequencing was isolated from the mycelia with an EZ1 DNA Tissue Kit and a Bio Robot EZ1 (Qiagen) according to the protocol for extraction of nucleic acid from Gram-positive bacteria. The size, purity, and double-strand DNA concentration of the genomic DNA were measured by pulsed-field gel electrophoresis, ratio of absorbance values at 260 nm and 280 nm, and Quant-iT PicoGreen dsDNA Assay Kit (Life Technologies) to assess the quality.

### Genome sequencing and assembly

Shotgun and pair-end libraries were prepared and sequenced using 454 pyrosequencing technology and HiSeq1000 (Illumina) pair-end technology, respectively (Table 2). The 70 Mb shotgun sequences and 702 Mb pair-end sequences were assembled into 20 scaffolds larger than 500 bp using Newbler v2.6, and subsequently finished using GenoFinisher [11].

### Genome annotation

Coding sequences were predicted by Prodigal [12] and tRNA-scanSE [13]. The gene functions were annotated using an in-house genome annotation pipeline and domains related to PKS and NRPS were searched for using the SMART and PFAM domain databases. PKS and NRPS gene clusters and their domain organizations were analyzed manually. Similarity search in the NCBI nr databases was also used for functional prediction of genes in the lydicamycin biosynthetic gene cluster.

## Genome properties

The total size of the genome is 8,319,549 bp and the GC content is 71.0 % (Table 3), similar to other genome-sequenced *Streptomyces* members. Of the total 7,344 genes, 7,240 are protein-coding genes and 75 are RNA genes. The classification of genes into COGs functional categories is shown in Table 4. As for the secondary metabolism, *Streptomyces* sp. TP-A0598 has two type I PKS, two type II PKS, two NRPS, and two hybrid PKS/NRPS gene clusters, suggesting the high capacity of production of polyketides and nonribosomal peptides.

**Table 3:** Genome statistics

Attribute	Value	% of Total
Genome size (bp)	8,319,549	100.0
DNA coding (bp)	7,149,098	85.9
DNA G + C (bp)	5,915,420	71.0
DNA scaffolds	20	100.0
Total genes	7,344	100.0
Protein-coding genes	7,240	98.6
RNA genes	75	1.0
Pseudo genes	29	0.4
Genes in internal clusters	761	10.4
Genes with functional prediction	3,207	43.7
Genes assigned to COGs	4,450	60.6
Genes with Pfam domains	4,543	61.9
Genes with signal peptides	653	8.9
Genes with transmembrane helices	1,770	24.1
CRISPR repeats	5	-

**Table 4:** Number of genes associated with general COG functional categories

Code	Value	% age	Description
J	196	2.70	Translation
A	2	0.03	RNA processing and modification
K	519	7.17	Transcription
L	155	2.14	Replication, recombination and repair
B	0	0.00	Chromatin structure and dynamics
D	40	0.55	Cell cycle control, mitosis and meiosis
V	127	1.75	Defense mechanisms
T	210	2.91	Signal transduction mechanisms
M	192	2.65	Cell wall/membrane biogenesis
N	0	0.00	Cell motility
U	34	0.47	Intracellular trafficking and secretion
O	138	1.91	Posttranslational modification, protein turnover, chaperones
C	271	3.74	Energy production and conversion
G	318	4.39	Carbohydrate transport and metabolism
E	424	5.86	Amino acid transport and metabolism
F	105	1.45	Nucleotide transport and metabolism
H	161	2.22	Coenzyme transport and metabolism
I	187	2.58	Lipid transport and metabolism
P	177	2.44	Inorganic ion transport and metabolism
Q	141	1.95	Secondary metabolites biosynthesis, transport and catabolism
R	631	8.72	General function prediction only
S	422	5.83	Function unknown
-	2,790	38.50	Not in COGs

The total is based on the total number of protein coding genes in the genome

## Insights from the genome sequence

**Fig. 4:**
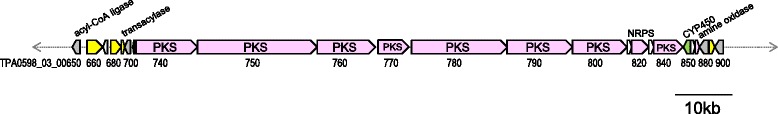
Genetic map of lydicamycin biosynthetic gene cluster

**Fig. 5:**
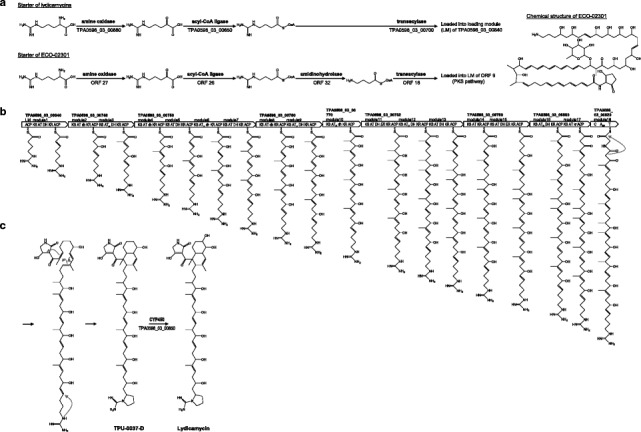
Proposed lydicamycin synthetic pathway. **a** starter synthesis compared with that of ECO-02301; **b** chain elongation; **c** cyclization and modification yielding final products

The chemical structure of lydicamycin (Fig. 1) suggests that its carbon skeleton is assembled from eleven malonyl-CoA and six methylmalonyl-CoA precursors by type I PKS pathway. In addition, this pathway should be combined with NRPS pathway since lydicamycin bears a tetramic acid moiety derived from the condensation of an amino acid to the polyketide chain. We therefore searched for a type I PKS gene cluster consisting of seventeen PKS modules and an NRPS module. A hybrid PKS/NRPS gene cluster in scaffold03 (Table 5, Fig. 4) consists of seventeen PKS modules and one NRPS module (Fig. 5b). According to the assembly line rule [14], the predicted structure of the polyketide arising from this PKS/NRPS hybrid gene cluster was in good accordance with the actual structure of lydicamycin (Fig. 5b). As a starter unit for the polyketide assembly, 4-guanidinobutyryl CoA could be proposed on the basis of annotation of TPA0598_03_00880, TPA0598_03_00650 and TPA0598_03_00700. These genes were predicted to encode amine oxidase, acyl-CoA ligase, and transacylase by comparing the corresponding genes present in the ECO-02301 biosynthetic gene cluster. In the biosynthesis of ECO-02301, 4-aminobutyryl-CoA is supplied from L-arginine by a sequential action of amine oxidase, acyl-CoA ligase, and amidinohydrolase and is transferred to ACP by transacylase (Fig. 5a) [15]. In the lydicamycin cluster, genes for an amine oxidase (TPA0598_03_00880), an acyl-CoA ligase (TPA0598_03_00650), and a transacylase (TPA0598_03_00700) are present in the surrounding region of the PKS cluster but an amidinohydrolase gene responsible for the hydrolysis of the guanidine residue to the primary amine is lacking (Fig. 5a, Table 5). After the 4-guanidinobutyryl starter is loaded onto ACP of TPA0598_03_00840, the polyketide chain is extended by eight PKSs and a glycine is added to the polyketide terminus by an NRPS module (Fig. 5b), followed by the formation of an octalin and a tetramic acid ring (Fig. 5c). It was not possible to assign a gene responsible for the cyclization of the guanidino precursor into a pyrrolidine ring. A cytochrome P450 (TPA0598_03_00850) would be responsible for the hydroxylation of the octalin carbon at C-8 (Fig. 5c). Production of deoxy- and demethylcongeners suggests that substrate recognition by the AT domain in module3 (second module of TPA0598_03_00740) and the ER domain in module11 (first module of TPA0598_03_00780) is likely not strict (Table 6).

**Table 5:** Open reading frames in the lydicamycin biosynthetic gene cluster

orf (locus tag)	size (aa)	proposed function	BLAST search	
			protein homolog, *origin,* accession number	%^b^
TPA0598_03_00650^a^	473	acyl-CoA ligase	hypothetical protein, *Streptomyces* sp. FxanaC1, WP_018093236	94/96
TPA0598_03_00660	929	LuxR family transcriptional regulator	LuxR family transcriptional regulator, *Streptomyces* sp. FxanaC1, WP_026170289	91/94
TPA0598_03_00670^a^	274	unknown	hypothetical protein, *Saccharomonospora azurea,* EHY88948	53/64
TPA0598_03_00680	632	two-component system histidine kinase	hypothetical protein, *Streptomyces* sp. FxanaC1, WP_018093233	93/95
TPA0598_03_00690	218	two-compornent system response regulator	LuxR family transcriptional regulator, *Streptomyces* sp. FxanaC1, WP_018093232	99/99
TPA0598_03_00700^a^	336	transacylase	ACP S-malonyltransferase, *Streptomyces* sp. FxanaC1, WP_026170288	89/93
TPA0598_03_00710^a^	123	unknown	hypothetical protein, *Streptomyces* sp. FxanaC1, WP_018093229	88/95
TPA0598_03_00720	64	unknown	hypothetical protein JCGZ_17256, *Jatropha curcas,* KDP45649	43/54
TPA0598_03_00730^a^	80	unknown	putative protein-disulfide isomerase, *Xanthomonas gardneri*, EGD16922	56/63
TPA0598_03_00740	3,598	PKS	polyketide synthase, *Streptomyces rapamycinicus,* AGP57755	58/69
TPA0598_03_00750	7,054	PKS	Beta-ketoacyl synthase, *Streptomyces violaceusniger,* AEM87320	57/68
TPA0598_03_00760	3,548	PKS	Beta-ketoacyl synthase, *Streptomyces violaceusniger,* AEM87320	56/67
TPA0598_03_00770	1,846	PKS	Beta-ketoacyl synthase, *Streptomyces iranensis,* CDR09758	62/73
TPA0598_03_00780	5,648	PKS	polyketide synthase type I, *Streptomyces aizunensis,* AAX98191	58/69
TPA0598_03_00790	3,662	PKS	hypothetical protein, *Streptomyces* sp. FxanaC1, WP_018091594	94/96
TPA0598_03_00800	3,265	PKS	polyketide synthase, *Streptomyces* sp. PRh5, EXU66032	54/66
TPA0598_03_00810	270	unknown	hypothetical protein, *Streptomyces* sp. FxanaC1, WP_018091596	95/96
TPA0598_03_00820	1,031	NRPS	hypothetical protein, *Streptomyces* sp. FxanaC1, WP_018091598	94/96
TPA0598_03_00830	300	unknown	hypothetical protein, *Streptomyces* sp. FxanaC1, WP_018091598	96/98
TPA0598_03_00840	1,923	PKS	hypothetical protein, *Streptomyces* sp. FxanaC1, WP_018091599	91/94
TPA0598_03_00850^a^	429	cytochrome P450	cytochrome P450, *Streptomyces* sp. FxanaC1, WP_026169967	92/96
TPA0598_03_00860	260	unknown	membrane protein, *Saccharopolyspora rectivirgula,* KEI45939	45/69
TPA0598_03_00870	253	type-II thioesterase	hypothetical protein, *Streptomyces* sp. FxanaC1, WP_018091603	95/97
TPA0598_03_00880^a^	551	amine oxidase	amine oxidase, *Streptomyces* sp. FxanaC1, WP_026169968	96/98
TPA0598_03_00890	344	transcriptional regulator	hypothetical protein, *Streptomyces* sp. FxanaC1, WP_018091605	96/97
TPA0598_03_00900^a^	496	amidase	hypothetical protein, *Streptomyces* sp. FxanaC1, WP_018091606	94/95

^a^encoded in complementary strand, ^b^identity/similarity

**Table 6:** Proposed mechanism to produce lydicamycin congeners

congener	substrate of m3 *AT*_*m*_	m11 *ER*	CYP450
lydicamycin	methylmalonyl-CoA	active	involved
TPU-0037-A	malonyl-CoA	active	involved
TPU-0037-B	methylmalonyl-CoA	inactive	uninvolved
TPU-0037-C	malonyl-CoA	active	uninvolved
TPU-0037-D	methylmalonyl-CoA	active	uninvolved

## Conclusions

The 8 Mb draft genome of *Streptomyces* sp. TP-A0598, a producer of lydicamycins isolated from seawater, has been deposited at GenBank/ENA/DDBJ under accession number BBNO00000000. We successfully identified the PKS/NRPS hybrid cluster for lydicamycin biosynthesis and proposed a plausible biosynthetic pathway. In addition, the genome of strain TP-A0598 contained seven orphan PKS or NRPS gene cluster but secondary metabolites from these orphan clusters have not been isolated yet. The genome sequence information disclosed in this study will be utilized for the investigation of additional new bioactive compounds from this strain and will also serve as a valuable reference for evaluation of the metabolic potential in marine-derived *Streptomyces*.

## Abbreviations

A_gly_: Adenylation domain whose substrate is glycine
ACP: Acyl carrier protein domain
AT: Acyltransferase domain whose substrate is malonyl-CoA
AT_m_: AT whose substrate is methylmalonyl-CoA
C: Condensation domain
CoA: Coenzyme A
CYP450: Cytochrome P450
DH: Dehydratase domain
dh: Inactive DH
ER: Enoylreductase domain
ISP: International *Streptomyces* project
KS: Ketosynthase domain
KR: Ketoreductase domain
kr: Inactive KR
LM: Loading module
m: Module
MRSA: Methicillin-resistant *Staphylococcus aureus*
NRPS: Nonribosomal peptide synthetase
PKS: Polyketide synthase
T: Thiolation domain
TE: Thioesterase domain
